# Augmenting autophagy for prognosis based intervention of COPD-pathophysiology

**DOI:** 10.1186/s12931-017-0560-7

**Published:** 2017-05-04

**Authors:** Manish Bodas, Neeraj Vij

**Affiliations:** 1 Molecular & Cell Biology, College of Medicine, Central Michigan University, Mt Pleasant, 2630 Denison Drive, Room# 120 (Office) & 126-127 (Lab), College of Medicine Research Building, Mt. Pleasant, MI 48859 USA; 2 Department of Pediatrics and Pulmonary Medicine, The Johns Hopkins University School of Medicine, Baltimore, Maryland USA

**Keywords:** Tobacco, Nicotine, Cigarette, e-cigarette, COPD, Emphysema, ROS, Oxidative stress, Autophagy

## Abstract

Chronic obstructive pulmonary disease (COPD) is foremost among the non-reversible fatal ailments where exposure to tobacco/biomass-smoke and aging are the major risk factors for the initiation and progression of the obstructive lung disease. The role of smoke-induced inflammatory-oxidative stress, apoptosis and cellular senescence in driving the alveolar damage that mediates the emphysema progression and severe lung function decline is apparent, although the central mechanism that regulates these processes was unknown. To fill in this gap in knowledge, the central role of proteostasis and autophagy in regulating chronic lung disease causing mechanisms has been recently described. Recent studies demonstrate that cigarette/nicotine exposure induces proteostasis/autophagy-impairment that leads to perinuclear accumulation of polyubiquitinated proteins as aggresome-bodies, indicative of emphysema severity. In support of this concept, autophagy inducing FDA-approved anti-oxidant drugs control tobacco-smoke induced inflammatory-oxidative stress, apoptosis, cellular senescence and COPD-emphysema progression in variety of preclinical models. Hence, we propose that precise and early detection of aggresome-pathology can allow the timely assessment of disease severity in COPD-emphysema subjects for prognosis-based intervention. While intervention with autophagy-inducing drugs is anticipated to reduce alveolar damage and lung function decline, resulting in a decrease in the current mortality rates in COPD-emphysema subjects.

## Background

All cellular systems maintain a homeostatic state that forms the foundation of a normal healthy lifestyle, although modest changes in homeostatic processes by genetic, environmental exposure, aging and/or unhealthy eating habits can result in a disease state. Out of the several complex and fascinating cellular homeostatic mechanisms, ‘autophagy’, is a key cellular degradation system, which can be modulated by any of the factors described above, resulting in chronic disease pathogenesis [1, 2]. Thus, the recent upsurge in chronic diseases is attributed to dramatic changes in lifestyle, where environmental factors (smoke, poor eating habits, lack of exercise etc.), in addition to genetic or polymorphic changes are considered to be the key mediators of disease initiation or progression. Among these debilitating chronic conditions, COPD (chronic obstructive pulmonary disease) is currently the 3rd leading cause of death [3], although it is preventable by making early lifestyle changes such as completely avoiding tobacco smoking or exposure. It is well known that first and second hand cigarette smoke exposure is the leading cause of COPD initiation and progression [4], while other contributing risk factors are respiratory infections, air pollution, exposure to biomass-smoke or e-cig/nicotine-vapors, aging, certain genetic polymorphisms and obesity [4–9]. The continuous exposure of our airways to the plethora of noxious agents present in tobacco smoke and/or e-cig/nicotine vapors initiates inflammatory-oxidative stress that leads to the eventual irreversible damage of the lung parenchyma and alveolar walls [4, 10–12]. Clinically, this progresses to alveolar emphysema, which is the primary pathological feature of COPD and is known to worsen with the age [4, 13]. Thus, early detection of emphysematous changes would significantly assist in the timely head start of the currently available treatments such as corticosteroids, bronchodilators or the proposed autophagy augmentation strategy. Although, recent advances in emphysema detection methods and research aimed towards developing novel biomarkers has improved the possibility of early emphysema detection and therapeutic intervention, these techniques still lack the required accuracy and high-throughput screening capabilities [14]. Thus, clinical prognosis of COPD-emphysema still heavily relies on pulmonary function testing using spirometry [15], and no laboratory based precise diagnostic test exists for the early detection of COPD-emphysema initiation, especially in subjects who are smokers without any noticeable signs of lung function decline [16], such as shortness of breath (or dyspnea). Although PFT/spirometry are quite robust in detection of airflow obstruction, they lack the sensitivity to quantify minimal early changes in pulmonary function [14]. Moreover, PFT procedure needs complete patient support that is sometimes unachievable especially in a sick subject. These hurdles warrant an urgent need for alternative screening techniques for better prognosis based intervention of COPD-emphysema.

**Fig. 1:**
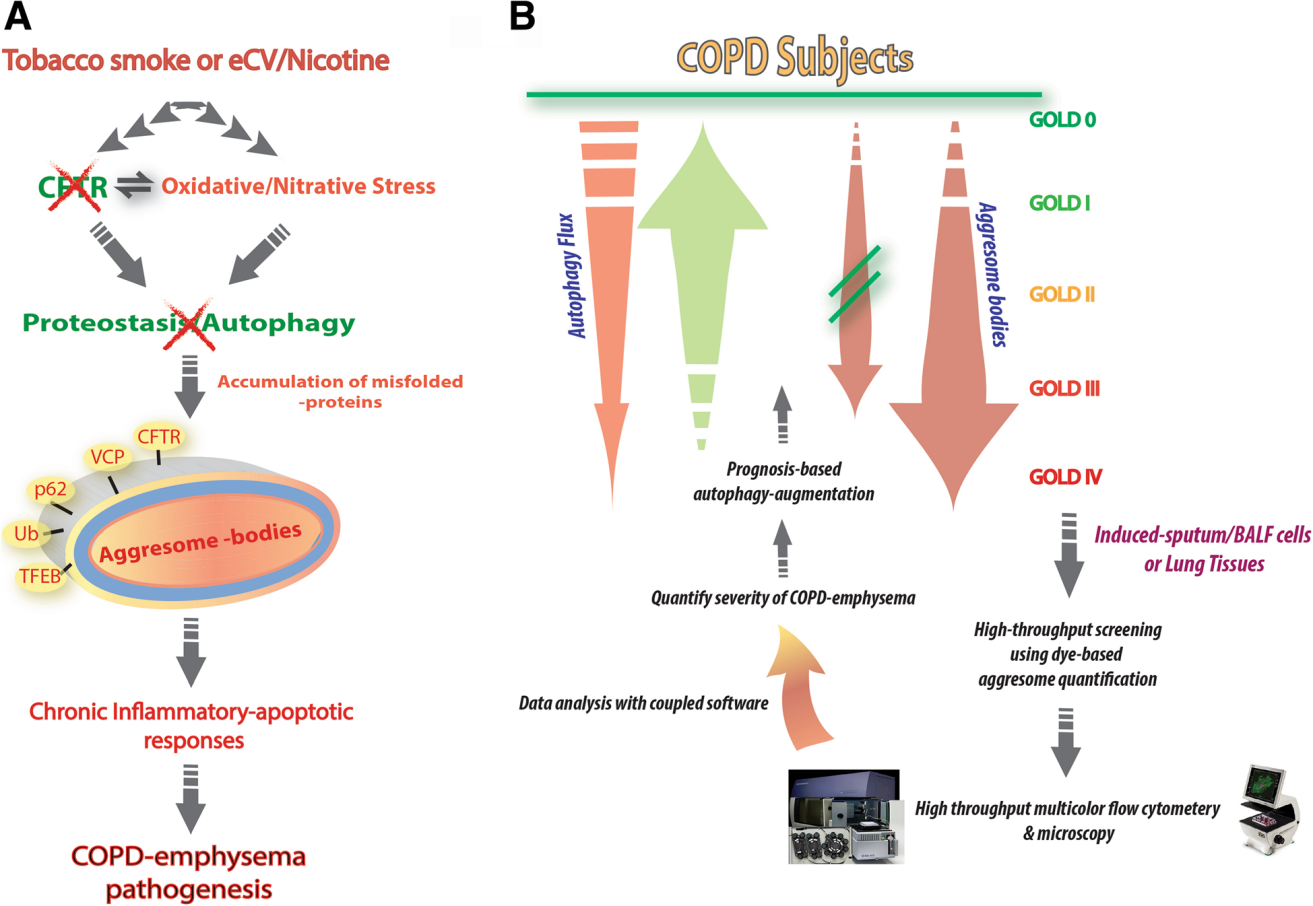
Rationale and design of a novel prognosis-based intervention strategy for COPD-emphysema. **a** Exposure to tobacco, e-cigarette vapor (eCV) or nicotine leads to oxidative-nitrative stress that mediates autophagy-impairment initiating aggresome formation, which acts as a central mechanism regulating COPD-emphysema pathogenesis. Thus, aggresome-bodies are implicated in triggering multifarious pathogenic mechanisms such as chronic inflammatory-apoptotic responses that drives the initiation and progression of emphysema in COPD subjects. **b** The proposed application of a non-invasive high throughput screening methodology for detecting aggresome-bodies in the cells derived from induced-sputum or bronchoalveolar lavage fluid (BALF/or lung biopsy sections) to predict COPD-like symptoms in non-smokers or smokers without any clinical signs of the lung disease. The high throughput flow cytometry and microscopy will assist in rapid screening of multiple samples for the presence and quantification of aggresome-bodies. The data generated from such high throughput assay will be analyzed by coupled software that assists in determining the severity of aggresome pathology and COPD-emphysema lung disease. This will allow categorization of subjects into different stages of the disease, based on the levels of aggresomes, which statistically correlates with the lung function decline and COPD-emphysema GOLD (Global Initiative for Chronic Obstructive Lung Disease) stage. Furthermore, the proposed prognosis-based personalized intervention strategy will utilize autophagy augmentation based on the levels of aggresomes that quantifies both the extent of autophagy-impairment and lung function decline. Overall, timely detection and treatment of emphysema or lung function decline by proposed prognosis based intervention strategy will help reduce current mortality rates in this fatal lung condition

Emerging data from our group and others have remarkably improved our understanding of the autophagy process in relation to its central role in the progression of COPD-emphysema [10–12, 17–22]. Exposure to tobacco and/or e-cig/nicotine vapor elevates oxidative-nitrative stress and inflammation that results in autophagy-flux impairment, further hampering the vital cellular homeostatic processes involved in the clearance of misfolded proteins and bacterial/viral pathogens that eventually impact the cell survival [10, 11, 18, 23–25]. Moreover, impaired autophagy flux results in perinuclear aggresome-bodies that are the hallmark of several neurodegenerative and protein-misfolding disorders [26–28]. The recent exhaustive studies using variety of pre-clinical models that includes human lung structural cells or tissues have clearly demonstrated that tobacco-smoke impaired-autophagy mediates accumulation of aggresome-bodies, which is primarily a cytoplasmic organelle comprised of aggregated (misfolded or damaged) proteins that initiates chronic inflammatory-apoptotic responses leading to senescence and emphysema progression (Fig. 1a) [10, 11, 17, 18, 20, 22, 25, 29, 30]. The pathogenic role of autophagy-impairment in COPD-emphysema is supported by findings from multiple studies demonstrating the accumulation of ubiquitinated proteins and p62 (an impaired-autophagy marker) that accelerates cellular senescence and COPD-emphysema pathogenesis [10, 11, 17, 18, 20, 22, 25, 29, 30]. Moreover, even acute exposure to cigarette smoke (CS) leads to elevated inflammatory-oxidative stress-mediated cellular apoptosis that statistically and functionally correlates with an accumulation of misfolded poly-ubiquitinated proteins as aggresome-bodies (insoluble protein fraction) in alveolar epithelial or structural cells and lungs (human and murine) [10, 11, 17, 18, 20, 22, 25, 29, 30]. Although acute CS exposure initiates modest changes in autophagy [31–33] but with chronic exposures autophagy-flux is impaired [10, 12]. The data showing the constitutive augmentation of basal airspace enlargement (emphysema) in LC3-knockout mice (intrinsically deficient in autophagy) [32] also supports these conclusions as impaired-autophagy drives both COPD-emphysema initiation and progression, although results were obviously interpreted inaccurately in this initial study due to the lack of impaired autophagy markers (such as p62) or autophagy-flux quantification. Thus, it is crucial to investigate the autophagy process in its entirety, which is collectively termed as ‘autophagy-flux’, and mere detection of autophagosomes and the quantification of LC3 processing is an inadequate and misleading methodology for the accurate evaluation of the autophagy response against external insults such as chronic CS [12, 34]. In addition, recent studies also emphasize the role of mitophagy, a form of autophagy that selectively degrades dysfunctional mitochondria, in COPD-emphysema [29, 35–37]. It is suggested that CS-induced mitochondrial-dysfunction [29, 36–38] and insufficient mitophagy collectively contributes to lung cellular senescence and progression of COPD [39], while augmenting mitophagy in human lung fibroblasts reduces or inhibits accumulation of damaged mitochondria and the resulting cellular senescence, suggesting a therapeutic benefit in COPD subjects [39].

Thus, we discuss here the twofold benefits of using aggresome-bodies as a prognostic marker for predicting emphysema initiation or progression in COPD subjects, in order to develop a potent prognosis-based intervention strategy utilizing autophagy augmentation, for the early diagnosis to allow prevention or reversal of COPD-emphysema related aggresome/lung pathologies (Fig. 1b).

### Rationale for targeting autophagy in COPD-emphysema

There is a strong rationale behind the hypothesis that augmenting autophagy could be a clinically relevant mode of controlling the early-onset of COPD-emphysema symptoms. The advent of COPD-emphysema is multifactorial that initiates from tobacco smoke exposure or aging-mediated alterations in several cellular homeostatic processes [13, 17, 24, 40–43], which eventually lead to late-stage symptoms such as severe lung function decline, indicative of fatal [44] or irreversible emphysema (GOLD III/IV). This is validated by recent studies, which demonstrate that tobacco-smoke (CS, eCV or nicotine) exposure, and aging induces ROS/RNS activity mediated autophagy-impairment as a central pathogenic mechanism for initiating chronic inflammatory-oxidative stress and apoptotic/senescent responses resulting in emphysema pathogenesis and progression. In fact, these functional changes statistically correlate with the severity of emphysema in COPD subjects [10–12, 17, 18, 43], verifying their role in lung disease progression. Mechanistically, the role of CS-induced acquired CFTR-dysfunction in mediating inflammatory-oxidative stress and the resulting autophagy-impairment as the key regulator of COPD-emphysema and obstructive lung pathologies is well described [12, 45, 46]. In addition, our recent study shows the statistical correlation of COPD-emphysema severity with aggresome-sequestration of transcription factor-EB (TFEB, the master regulator of autophagy and lysosomal biogenesis genes) in structural cells such as bronchial and alveolar epithelial [10]. Moreover, further experimental analysis demonstrates that functional knockdown of TFEB results in elevated oxidative stress, autophagy-flux impairment (aggresome-formation), cellular apoptosis and senescence in bronchial epithelial cells [10], similar to the effects of tobacco smoke exposure or aging [43].

Apart from the structural cells in the airway, the innate and adaptive immune cells also play a vital role in the immunopathogenesis of CS/age-related COPD-emphysema [20, 47–49]. The airways of COPD patients are populated with several types of immune cells such as macrophages, neutrophils, T-lymphocytes and dendritic cells, which collectively mediate the hyper-inflammatory state observed in COPD lungs [47]. These inflammatory cells and their secretory products (such as cytokines, chemokines and proteases) are the major contributors of lung parenchymal destruction that results in emphysematous changes, which is the major characteristic of CS/age-related COPD [47]. Several studies highlight the critical role of autophagy in regulating the biogenesis as well as function of immune cells such as T cells, B cells, dendritic cells, macrophages etc. [50, 51]. Specifically, recent studies have demonstrated the critical role of autophagy in regulating ‘inflammasome’ activation [52, 53] and release of pro-inflammatory cytokines such as IL-1β [51, 54] that contributes to initiation of hyper-inflammatory response(s) in COPD-emphysema [55, 56]. Mechanistically, loss of autophagy in immune cells such as macrophages or dendritic cells leads to ROS-mediated inflammasome and pro-inflammatory response activation via NFκB, IL-1α, IL-1β and IL-18 etc. [52]. Additionally, impairment of autophagy is also associated with an increase in ROS-dependent macrophage migration inhibitory factor (MIF) activity [57], which further promotes pro-inflammatory cytokine (such as IL-1β) production and release [54], as well as hyper-inflammatory response in COPD airways [58]. In addition, as a proof of concept, macrophages isolated from COPD subjects are defective in autophagy-mediated phagocytosis (xenophagy) resulting in impaired clearance of bacteria/viruses [59–61]. This is further supported by recent findings from our group on the role of tobacco smoke/nicotine-mediated autophagy-impairment in bacterial clearance in experimental models of COPD [11, 23].

Thus, comprehensive analysis of multiple studies presents significant evidence justifying the central role of proteostasis- and autophagy- impairment in COPD-emphysema pathogenesis [10–12, 18–20, 22, 24, 25], as the rationale for its augmentation is to controllung disease progression in COPD-emphysema subjects. In addition, changes in autophagy-flux and aggresome-pathology can serve as an early prognostic marker for predicting emphysema initiation or progression.

### The perspective on aggresome-pathology as a prognostic marker for COPD-emphysema

We and others have extensively documented the negative impact of tobacco smoke and/or eCV/nicotine exposure on the proteostasis and autophagy machinery that leads to the formation of perinuclear aggregates of polyubiquitinated proteins, ‘aggresomes’ [10, 11, 17–19]. Formation of aggresomes is perceived as a cytoprotective mechanism in response to cellular stimulus such as inflammatory-oxidative stress [62]. The inability of the cell to efficiently clear-off these pathogenic protein aggregates induced by CS-mediated proteostasis and autophagy impairment [63], results in premature senescence and initiation of emphysema-related pathogenic manifestations such as chronically elevated inflammatory-apoptotic responses that result in alveolar airspace enlargement [10, 19, 22, 64, 65]. As a proof-of-concept, transgenic mice with diminished proteasomal activity are in fact susceptible to CS-induced emphysematous lung destruction [25].

We have used different cellular markers for the detection and quantification of impaired-autophagy such as accumulation of ubiquitinated proteins (Ub), p62 and valosin containing protein (VCP), in in vitro (CSE treated structural/epithelial cells) and in vivo (mice exposed to CS) models of emphysema, as well as human lung tissues from COPD-emphysema subjects [10, 12, 17–19]. These data helped us to reveal aggresome-bodies (p62-accumulation) as a predictive biomarker of lung function decline and emphysema severity in COPD subjects (GOLD 0-IV) [10, 12, 17]. Moreover, this severity-related increase in accumulation of p62- and aggresome-bodies- was not observed in non-smoking subjects, validating our hypothesis that CS-induced aggresome-formation is driven by autophagy-impairment that mediates COPD-emphysema pathogenesis [17]. Thus, as discussed above, it is important to use co-localization of Ub with p62 (or LC3β) to evaluate the autophagy-flux levels, or quantify the aggresome-bodies by a dye-based bioassay, as a predictive marker of COPD-emphysema severity [10, 12] as proposed here. The need for such predictive disease biomarker is elevated, due to the recent exponential rise in the use of e-cigarettes that are marketed as a safer alternative to regular cigarettes. As a proof-of-concept, we demonstrated that autophagy-flux and aggresome-bodies can serve as an early predictive marker of e-cigarette vapor (eCV) induced cellular senescence in airway structural/epithelial cells that drives COPD-emphysema pathogenesis [18]. To the dismay of many e-cig supporters, our study identified that even acute-eCV/nicotine exposure leads to aggresome-formation through proteostasis and autophagy impairment that mediates chronic inflammatory-apoptotic response that can eventually induce senescence and COPD-emphysema in these subjects on chronic exposures [11, 18]. In support of this data, a recent study shows that chronic e-cig exposure in mice triggers COPD pathophysiology in a nicotine-dependent manner [66]. Thus, these findings further assert that e-cig/nicotine or waterpipe smoke (WPS) exposure can initiate the chronic inflammatory-apoptotic responses and senescence via ROS mediated autophagy-impairment, potentially leading to COPD-emphysema development [11, 67]. Evidently, even acute eCV exposure of human subjects leads to changes in markers of COPD such as exhaled nitric oxide (eNO) [68]. Collectively, these thorough and multiple studies highlight our perspective that quantification of aggresome-bodies has a potential translational application in predicting the COPD-emphysema disease severity that can assist in designing a personalized prognosis-based intervention strategy.

To this end, the recent studies utilize a novel dye-based aggresome quantification bioassay [18, 25] for predicting COPD-emphysema. This non-invasive flow cytometry or microscopy based aggresome quantification assay can either utilize induced sputum or bronchoalveolar lavage fluid (BALF) cells, and/or the lung tissue biopsy﻿ samples (for confirmation if available) from the human subjects [10, 12, 18]. Thus, this dye-based aggresome quantification assay has the potential to be developed as a prognostic tool for high-throughput early non-invasive screening of COPD subjects using the induced-sputum or BALF cells for flow cytometry or microscopy based aggresome quantification as an early predictor of emphysema severity.

### Prognosis based intervention strategy for COPD-emphysema

The induced-sputum or BALF cells collected from the prospective human subjects uses sputum-induction [69] or bronchoscopy [70], and the cells are processed for immunostaining as recently described [71]. Briefly, cells stained with a specific aggresome dye (red) are quantified by a high-throughput flow cytometry and microscopy that assist in the acquisition and analysis of the induced-sputum/BALF cell population [71]. Thus, quantification of the number of aggresome-positive cells in a subject will assist us in determining the severity of COPD-emphysema, as discussed above in detail. This would allow a prognosis-based therapeutic intervention using autophagy inducing drugs based on the levels of aggresome-bodies predictive of the disease state of the particular subject (Fig. 1b).

The utility of proteostasis and autophagy inducing compounds in restoring CS-induced COPD-emphysema progression is apparent based on the therapeutic potency of clinically safe FDA approved autophagy-inducing anti-oxidant drugs (such as cysteamine, carbamazepine, gemfibrozil and fisetin), in the in vitro and preclinical COPD-emphysema models [10–12, 18]. Moreover, recent studies have demonstrated the clinical potential of these drugs in variety of chronic obstructive lung diseases, where insufficient-autophagy has been shown to mediate pathogenesis of lung diseases such as cystic fibrosis (CF) [72, 73], asthma [74] and idiopathic pulmonary fibrosis (IPF) [75, 76], further supporting the therapeutic potential of autophagy-augmenting drugs in COPD-emphysema. In fact, these drugs showed an excellent control of tobacco smoke or eCV/nicotine exposure induced aggresome-pathology [10–12, 18] thus allowing a wider prospective therapeutic window that we aim to utilize to control COPD-emphysema disease progression. One of the leading candidate for autophagy inducing drug is cysteamine, based on its well-characterized properties as a potent anti-oxidant, bactericidal and mucolytic, which is currently under clinical evaluation (procysbi, cystagon or lynovex) for the treatment of other chronic diseases such as CF [77–79]. In addition, the CFTR-rescuing property of cysteamine [78, 80] will provide additional therapeutic advantage in COPD by controlling CS-induced acquired CFTR-dysfunction [12, 45]. Briefly, the utility of cysteamine is promising but requires: (i) cautious standardization of the dose, as significantly higher doses have been reported to block autophagy-flux in the late stages of the autophagy process [81]; and (ii) a delivery method, as it’s in vivo efficacy is hampered by a short half-life, thus necessitating the development of suitable delivery systems for targeted and sustained drug delivery of a lower dose of an effective drug formulation through the mucus-dense airways of COPD subjects [80, 82]. Thus, we are currently developing autophagy-inducing compounds and their nano/dendrimer-based formulations for the intranasal (inhalation route) delivery of long half-life, stable and effective drug. This prospective strategy as discussed in detail elsewhere [82], will not only allow better distribution in the COPD-emphysema airways but will also help in retaining multiple therapeutic benefits of cysteamine, as an anti-oxidant, bactericidal and mucolytic [72, 83].

In summary, we propose a novel prognosis-based aggresome quantification bioassay for early diagnosis of emphysema for avoiding chronic progression of COPD-emphysema related aggresome/lung pathologies, where reverting or rescuing the lung parenchyma is not clinically feasible or difficult. Thus, the proposed strategy will not only prevent the progression of COPD-emphysema to severe stages but also provide a window of opportunity to either restrict or reverse the lung disease. We would like to emphasize here that reverting COPD-emphysema from chronic disease stages is not feasible unless tissue regeneration is utilized in parallel. Therefore, the proposed bioassay, which uses induced-sputum/BALF cells or the lung tissue biopsy samples from prospective or current COPD-emphysema subjects, will allow prognosis-based early intervention (via autophagy augmentation) for prevention of progressive chronic obstructive lung disease.

## Conclusion

COPD-emphysema is a “non-reversible” lung condition with fatal affects that are compounded by the lack of accurate and timely diagnosis. Hence, we present here a novel aggresome dye-based bioassay to allow early diagnosis of emphysema before its progression to severe stages. The proposed bioassay can utilize non-invasive induced-sputum cells, or bronchoscopy-directed BALF cells or the lung tissue biopsy sample collection from the human subjects to allow prognosis-based early intervention by an autophagy augmentation strategy for controlling the progression of the severe emphysematous lung disease.

## Abbreviations

BALF: Bronchoalveolar lavage fluid
CFTR: Cystic fibrosis transmembrane conductance regulator protein
COPD: Chronic obstructive pulmonary disease
CS: Cigarette smoke
CSE: Cigarette smoke extract
CYS: Cysteamine
eCV: e-cigarette vapor
eNO: Exhaled nitric oxide
FIS: Fisetin
GEM: Gemfibrozil
GOLD: Global Initiative for Chronic Obstructive Lung Disease
p62/SQSTM1: Sequestosome 1
PFT: Pulmonary function test
ROS: Reactive oxygen species
TFEB: Transcription factor-EB
Ub: Ubiquitin
VCP: Valosin containing protein
WPS: Waterpipe smoke
